# Association between Polyphenol Intake and Gastric Cancer Risk by Anatomic and Histologic Subtypes: MCC-Spain

**DOI:** 10.3390/nu12113281

**Published:** 2020-10-26

**Authors:** María Rubín-García, Facundo Vitelli-Storelli, Antonio José Molina, Raúl Zamora-Ros, Nuria Aragonés, Eva Adarnaz, Gemma Castaño-Vinyals, Mireia Obón-Santacana, Inés Gómez-Acebo, Ana Molina-Barceló, Guillermo Fernández-Tardón, José J Jiménez-Moleón, Juan Alguacil, María Dolores Chirlaque, Estefanía Toledo, Beatriz Pérez-Gómez, Marina Pollán, Manolis Kogevinas, Vicente Martín

**Affiliations:** 1 Group of Investigation in Interactions Gene-Environment and Health (GIIGAS); Institute of Biomedicine (IBIOMED), University of León, 24071 León, Spain; mrubig@unileon.es (M.R.-G.); ajmolt@unileon.es (A.J.M.); vicente.martin@unileon.es (V.M.); 2 Unit of Nutrition and Cancer, Cancer Epidemiology Research Programme, Catalan Institute of Oncology (ICO), Bellvitge Biomedical Research Institute (IDIBELL), L’Hospitalet del Llobregat, 08908 Barcelona, Spain; raulzamoraros@gmail.com; 3 Department of Health of Madrid, Epidemiology Section, Public Health Division, 28035 Madrid, Spain; nuria.aragones@salud.madrid.org; 4 Consortium for Biomedical Research in Epidemiology & Public Health (CIBER en Epidemiología y Salud Pública-CIBERESP), 28029 Madrid, Spain; me.ardanaz.aicua@cfnavarra.es (E.A.); gemma.castano@isglobal.org (G.C.-V.); ines.gomez@unican.es (I.G.-A.); fernandeztguillermo@uniovi.es (G.F.-T.); jjmoleon@ugr.es (J.J.J.-M.); alguacil@dbasp.uhu.es (J.A.); mdolores.chirlaque@carm.es (M.D.C.); bperez@isciii.es (B.P.-G.); mpollan@isciii.es (M.P.); manolis.kogevinas@isglobal.org (M.K.); 5 Institute for Health Research (IdiSNA), 31008 Pamplona, Spain; etoledo@unav.es; 6 Barcelona Institute for Global Health (ISGlobal), 08036 Barcelona, Spain; 7 Hospital del Mar Medical Research Institute (IMIM), 08003 Barcelona, Spain; 8 Department of Public Health, Universitat Pompeu Fabra (UPF), Campus del Mar, 08002 Barcelona, Spain; 9 ONCOBELL Program, Bellvitge Biomedical Research Institute (IDIBELL), L’Hospitalet De Llobregat, 08908 Barcelona, Spain; mobon@iconcologia.net; 10 Oncology Data Analytics Program (ODAP), Catalan Institute of Oncology (ICO), L’Hospitalet Del Llobregat, 08908 Barcelona, Spain; 11 Facultad de Medicina, Universidad de Cantabria; IDIVAL, 39011 Santander, Spain; 12 Cancer and Public Health Area, FISABIO-Public Health, 46020 Valencia, Spain; molina_anabar@gva.es; 13 Health Research Institute of the Principality of Asturias (ISPA), Oncology Institute, University of Oviedo, 33003 Oviedo, Asturias; 14 Instituto de Investigación Biosanitaria de Granada (ibs.GRANADA), Hospitales Universitarios de Granada; Universidad de Granada, 18071 Granada, Spain; 15 Centro de Investigación en Recursos Naturales, Salud y Medio Ambiente (RENSMA), Universidad de Huelva, Campus Universitario de El Carmen, 21071 Huelva, Spain; 16 Department of Epidemiology, Regional Health Council, IMIB-Arrixaca, Murcia University, Campus de Ciencias de la Salud, 30120 El Palmar, Murcia, Spain; 17 Centro de Investigación Biomédica en Red Fisiopatología de la Obesidad y la Nutrición (CIBEROBN), Institute of Health Carlos III, 28029 Madrid, Spain; 18 Department of Preventive Medicine and Public Health, University of Navarra, 31008 Pamplona, Spain; 19 Cancer and Environmental Epidemiology Unit, Department of Epidemiology and Chronic Diseases, National Center for Epidemiology, Carlos III Institute of Health, 28029 Madrid, Spain; 20 Cancer Epidemiology Research Group, Oncology and Hematology Area, IIS Puerta de Hierro, IDIPHIM, 28222 Madrid, Spain

**Keywords:** diet, epidemiology, gastric cancer, polyphenols, phenolic acids, stilbenes, lignans, anatomic, histologic, MCC-Spain

## Abstract

Several anticancer properties have been largely attributed to phenolics in in vivo and in vitro studies, but epidemiologic evidence is still scarce. Furthermore, some classes have not been studied in relation to gastric cancer (GC). The aim of this study was to assess the relationship between the intake of phenolic acids, stilbenes, and other phenolics and the risk of developing GC and its anatomical and histological subtypes. We used data from a multi-case-control study (MCC-Spain) obtained from different regions of Spain. We included 2700 controls and 329 GC cases. Odds ratios (ORs) were calculated using mixed effects logistic regression considering quartiles of phenolic intake. Our results showed an inverse association between stilbene and lignan intake and GC risk (OR_Q4 vs. Q1_ = 0.47; 95% CI: 0.32–0.69 and OR_Q4 vs. Q1_ = 0.53; 95% CI: 0.36–0.77, respectively). We found no overall association between total phenolic acid and other polyphenol class intake and GC risk. However, hydroxybenzaldehydes (OR_Q4 vs. Q1_ = 0.41; 95% CI: 0.28–0.61), hydroxycoumarins (OR_Q4 vs. Q1_ = 0.49; 95% CI: 0.34–0.71), and tyrosols (OR_Q4 vs. Q1_ = 0.56; 95% CI: 0.39–0.80) were inversely associated with GC risk. No differences were found in the analysis by anatomical or histological subtypes. In conclusion, a diet high in stilbenes, lignans, hydroxybenzaldehydes, hydroxycoumarins, and tyrosols was associated with a lower GC risk. Further prospective studies are needed to confirm our results.

## 1. Introduction

Gastric cancer (GC) is the third leading cause of cancer-related death in the world [1], and its 5-year survival after diagnosis is below 30% in many countries [2]. GC incidence rates vary across geographical areas, probably due to risk factors such as dietary patterns, lifestyle habits, genetics, and exposure to carcinogens [3].

Between 10% and 20% of GC patients have a family history—although only 1–3% of them show a clear Mendelian inheritance pattern—and 80% to 90% are considered sporadic [4]. Thus, environmental factors seem highly relevant for gastric carcinogenesis and, therefore, a large proportion of GC cases are potentially preventable.

More than 20% of deaths due to GC are attributed to obesity/overweight, 5% to physical inactivity, 5% to excessive alcohol consumption, and 5% to a poor diet [5], which are all related to lifestyles. Accordingly, fruit consumption has been identified as a protective factor against GC and this may partly be due to the fruit’s content of polyphenols (PLPs) [6].

PLPs are secondary metabolites of plants, which can be found in fruit, vegetables, cereals, and their derived beverages (such as coffee, tea, wine, and juices). These compounds have been shown to exert preventive properties against a wide range of chronic conditions, including diabetes, cardiovascular problems, neurodegenerative diseases, and cancer [7,8].

The anticancer properties of PLPs have been largely attributed to their great anti-inflammatory and antioxidant potential, as well as their ability to modulate signaling pathways and molecular targets. These mechanisms have been associated with cancer processes such as cell survival, differentiation, proliferation, migration, hormonal activities, angiogenesis, immune responses, or detoxifying enzymes [9].

Most of this knowledge has been obtained from in vitro studies, which have mainly focused on the flavonoid family [10]. Other PLP families, such as phenolic acids, stilbenes, lignans, and other phenolics, have been less studied; however, epidemiological evidence is still limited [11].

Since risk factors for GC are different depending on its anatomical and histological subtype [12,13], we hypothesize that the different classes of PLPs can be associated with different GC types. The aim of this study was to assess whether the intake of phenolic acids, stilbenes, lignans, and other PLPs was associated with the risk of developing GC, also according to its anatomical and histological subtypes.

## 2. Materials and Methods

### 2.1. Study Population

The multi-case-control (MCC)-Spain study is a multicenter, population-based case-control study that was carried out in 12 Spanish provinces to examine potential associations between environmental and genetic factors and the risk of five common cancers. Detailed information on the study design can be found elsewhere [14]. Cases and controls were recruited from 2008 to 2013 in 16 hospitals. The inclusion criteria for cases of histologically confirmed gastric tumors were having lived for at least six months in the area of the hospital and being between 20 and 85 years old. Controls were frequency matched to the overall distribution of cancer cases by age, sex, and region (province).

**Figure 1:**
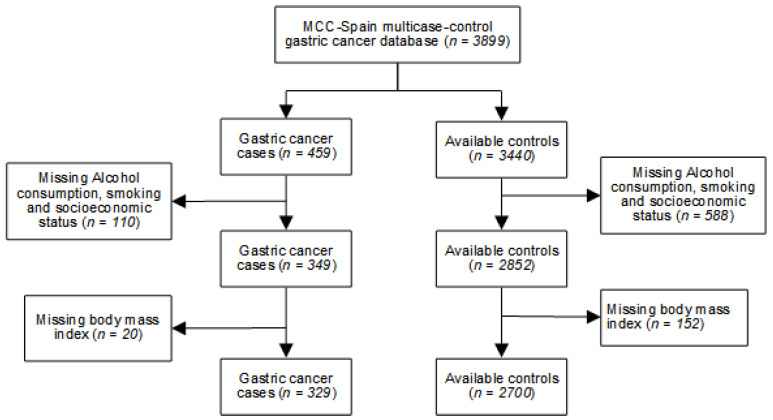
Flowchart of the participants’ selection in the multi-case-control (MCC)-Spain study.

For the specific case of GC, MCC-Spain recruited 459 cases of GC and 3440 controls. In the present study, 329 cases and 2700 controls were included after excluding participants with missing data (Figure 1).

All participants signed the informed consent after having been previously informed about the study. The study was designed according to the Declaration of Helsinki and the Spanish Data Protection Act 1999, and the ethics committees of the participating institutions approved the MCC-Spain study protocol [15].

### 2.2. Classification of Tumors

The pathology information and the rest of records obtained regarding the histology and anatomy of the GC were reviewed by qualified personnel. Collected clinical information for each gastric tumor case included anatomical subtype (cardia and non-cardia), extension, and histological subtype (intestinal and diffuse).

### 2.3. Variables and Data Collection

Cases and controls were interviewed by trained personnel at baseline. Information on sociodemographic factors, health behaviors (as physical activity or smoking), medical conditions and medical treatments, and family history of cancer was collected. In addition, cases and controls reported dietary habits, including current and past alcohol consumption (from 30 to 40 years old), mainly with a self-administered questionnaire.

#### 2.3.1. Assessment of Nutrient Intake

A validated food-frequency questionnaire (FFQ) [16] was collected at recruitment. The MCC-Spain dietary questionnaire includes questions about the previous year’s frequency of consumption of foods grouped under ten food categories: (1) Meat (products such as lamb, poultry, beef, pork, eggs, fish and seafood, and precooked meat-derived food), (2) legumes and vegetables, (3) nuts and fruits, (4) dairy products, (5) cereals (including bread and pasta), (6) seasonings and sauces, (7) oils and fats, (8) sweets and snacks, (9) vitamin and mineral supplements, (10) alcoholic and other beverages.

The daily consumption of each food was estimated based on reference tables of food servings. As in other studies, if a given food was a recipe (e.g., vegetable puree or gazpacho) the list of ingredients was calculated [17].

#### 2.3.2. Analysis of PLP Intake

The PLP classes considered in the present study were phenolic acids (including hydroxybenzoic acids, hydroxycinnamic acids, and hydroxyphenylacetic acids) stilbenes, lignans, and other phenolics (including alkylmethoxyphenols, methoxyphenols, hydroxycoumarins, tyrosols, and other minor phenolics).

For the analysis of PLP intake, a subset of 58 foods was considered, including legumes and vegetables, fruits, cereals, sweets and snacks, and alcoholic beverages and others. To estimate PLP intake, instead of considering the amount of all individual PLP chemical species (glycosides, esters, etc.) reported for different foods, we used aglycone equivalents. Dietary intake of the aglycone forms of PLP was estimated from the Phenol-Explorer database [18]. The rationale for this decision was to standardize data from different analytical methods to facilitate comparisons between studies [19].

PLP intake was calculated in milligrams per day, based on the food consumption data from the FFQ and the aglycone PLP content of each food referred in the Phenol-Explorer database. Estimation of the PLP contents involved obtaining the aglycone equivalents for each PLP subgroup and foods included in the Phenol-Explorer database. No retention factors were applied in the calculation of the amount of PLP intake.

### 2.4. Statistical Analysis

Descriptive statistics were used to display characteristics of cases and controls and by the tumor’s specific location and histology. Comparisons between cases and controls and between anatomical and histological subtypes were carried out using the Pearson chi square test (χ^2^) for categorical variables. Depending on the normality of the continuous variables, ANOVA or Kruskal-Wallis tests were used for qualitative traits. Significance for all statistical tests was set at *p* < 0.05.

PLP intake values were adjusted for energy intake [20] to estimate isocaloric intake of PLPs, separately for men and women.

PLP intake was categorized into quartiles according to the sample distribution among controls stratified by sex, and the lowest consumption category was always used as reference.

As additional analysis, PLP intake was transformed to log2, since the data were right-skewed [21]. This transformation had the same normalizing effect as the energy-adjusted by residual method. Its interpretation is simpler, given that the odds ratios (ORs) indicate the odds of GC when the intake is doubled (Supplementary Table S1).

Mixed-effects multivariate logistic regression models were performed to assess the association between PLP intake and cancer risk, including study area as a random effect term. Values are shown as OR and 95% confidence interval (CI). Other variables included in the multivariable model were age; sex; socioeconomic status (low, medium, and high); first-degree family history of GC; physical activity (as metabolic equivalent task (MET)-h/week); body mass index (BMI); smoking status; consumption of alcohol, vegetables, red meat, and salt; and total energy intake. Mixed-effects logistic regressions were performed with Stata statistical software release 13 [22]. Python version 3.14 [23] was used for the extraction of Phenol-Explorer web data on polyphenol content in foods, and R version 3.6 [24] was used for the calculation of the PLP intake by individuals.

## 3. Results

This study includes 329 GC cases and 2700 controls whose characteristics are described in Table 1. Cases were mostly male (72.6%) and had a higher percentage of family history of GC than that in controls (16.1 vs. 6.3). In addition, they presented a larger percentage of high alcohol drinkers and consumed on average more red meat (84.4 vs. 64.0 g/d) and more sodium (3529.3 vs. 3008.6 mg/g). The average consumption of the PLP subclasses of the entire sample is shown in Table 2, as well as main food sources for the different PLP subclasses.

**Figure 2:**
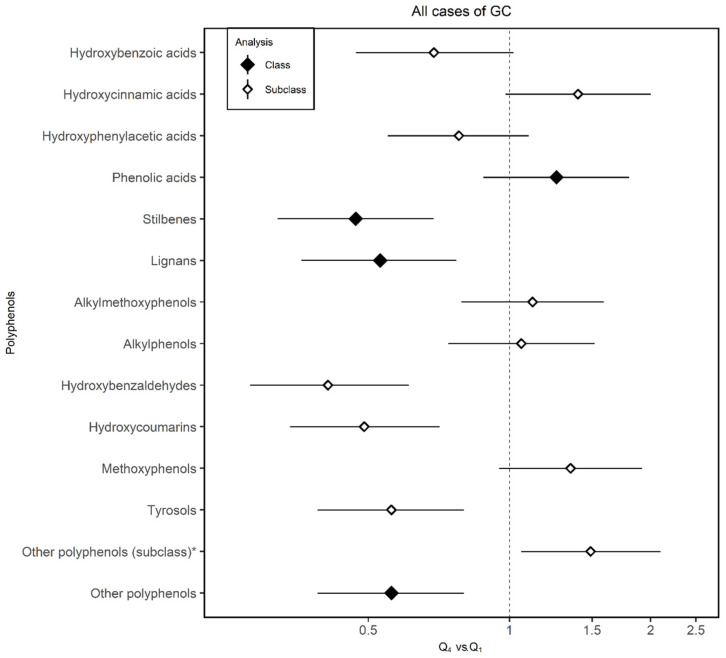
Adjusted odds ratios (ORs) and 95% confidence intervals (CIs) of gastric cancer (GC) for quartile 4 (Q4) vs. quartile 1 (Q1) of polyphenol intake in the MCC-Spain study. Estimated using unconditional logistic regression models adjusted for age; sex; socioeconomic status; smoking status; first-degree family history of GC; physical activity; body mass index; alcohol consumption; and vegetables, red meat, salt, and total energy intake including the study area as a random effect term. * Other polyphenols (subclass): Estimated ORs and their corresponding 95% confidence intervals for other polyphenols subclasses (including arbutin, catechol, coumestrol, phenol, phlorin, and pyrogallol).

Associations between the intake of phenolic classes and subclasses and the odds of developing GC are shown in Figure 2. Stilbenes were associated with a reduction in the odds of total GC by 53% (95% CI 0.32–0.69), lignans reduced the risk by 47% (95% CI 0.36–0.77), and hydroxybenzaldehydes by 59% (95% CI 0.28–0.61). The hydroxycoumarin subclass was associated with a 51% (95% CI 0.34–0.71) reduction in total GC risk, and tyrosols were associated with 44% lower odds of GC (95% CI 0.39–0.80). Regarding the consumption of other PLP subclass, the risk of total GC increased by 49% (95% CI 1.06–2.10). Hydroxybenzoic acids and hydroxyphenylacetic acids showed protective tendencies and hydroxycinnamic acids, methoxyphenols, and other PLPs (subclass) tended to increase the risk against GC although none of these associations were statistically significant.

**Figure 3:**
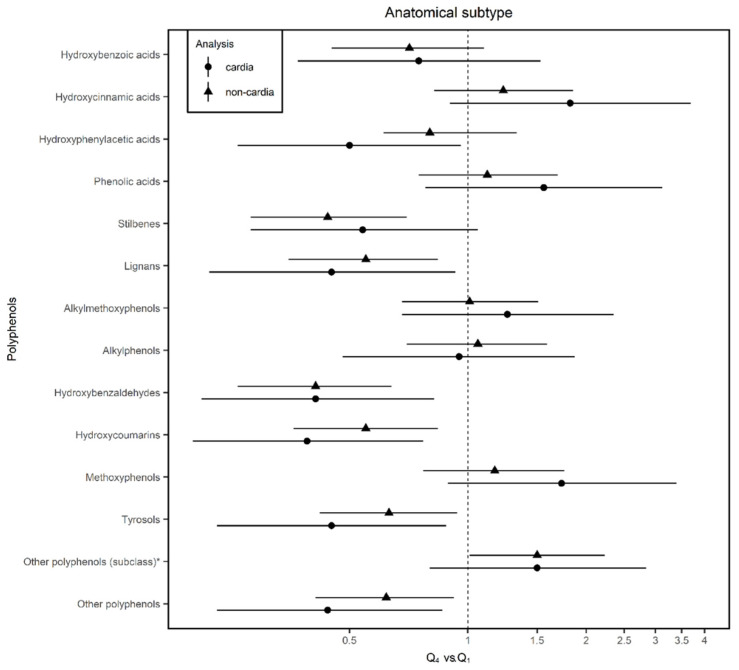
Adjusted odds ratios (ORs) and 95% confidence intervals (CIs) of gastric cancer (GC) by anatomical location for quartile 4 (Q4) vs. quartile 1 (Q1) of polyphenol intake in the MCC-Spain study. Estimated using unconditional logistic regression models adjusted for age; sex; socioeconomic status; smoking status; first-degree family history of GC; physical activity; body mass index; alcohol consumption; and vegetables, red meat, salt, and total energy intake including the study area as a random effect term. * Other polyphenols (subclass): Estimated ORs and their corresponding 95% confidence intervals for other polyphenols subclasses (including arbutin, catechol, coumestrol, phenol, phlorin, and pyrogallol).

When the association between the intake of PLP and GC was analyzed according to the anatomical site (Figure 3), results were similar to the results for GC risk. Hydroxyphenylacetic acids showed a 50% decrease (95% CI 0.26–0.96) in the risk of the cardia subtype, stilbenes of 56% (95% CI 0.28–0.70), and other PLP subclasses of 50% (95% CI 1.01–2.23) for non-cardia GC. Lignans were associated with a reduction in the odds of both subtypes, a 55% risk reduction (95% CI 0.22–0.93) in cardia GC and 45% reduction (95% CI 0.35–0.84) in non-cardia GC. Hydroxybenzaldehydes reduced the risk by 59% in cardia and non-cardia GC and hydroxycoumarin by 61% (95% CI 0.20–0.77) and 55% (95% CI 0.36–0.84), respectively. Finally, tyrosols were associated with a 61% (95% CI 0.20–0.77) reduction in cardia GC risk and 55% reduction (95% CI 0.36–0.84) in non-cardia GC risk.

**Figure 4:**
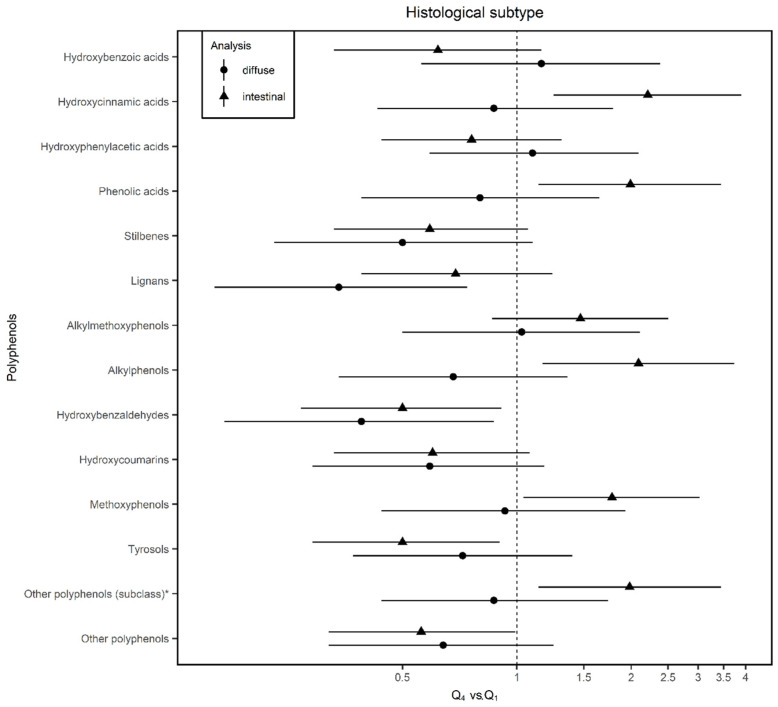
Adjusted odds ratios (ORs) and 95% confidence intervals (CIs) of gastric cancer (GC) by histological subtype for quartile 4 (Q4) vs. quartile 1 (Q1) of polyphenol intake in the MCC-Spain study. Estimated using unconditional logistic regression models adjusted for for age; sex; socioeconomic status; smoking status; first-degree family history of GC; physical activity; body mass index; alcohol consumption; and vegetables, red meat, salt, and total energy intake including the study area as a random effect term. * Other polyphenols (subclass): Estimated ORs and their corresponding 95% confidence intervals for other polyphenols subclasses (including arbutin, catechol, coumestrol, phenol, phlorin, and pyrogallol).

According to histological subtypes (Figure 4), we found some differences between PLP groups. For the intestinal subtype, hydroxycinnamic acids, alkylphenols, and other PLP classes, doubled the odds of GC risk; while methoxyphenols were associated with an increase of 78% in the odds of GC. Lignans were associated with a reduction of diffuse GC but were not associated with intestinal subtype. Hydroxybenzoic acids showed a non-significant tendency to reduce the odds of intestinal GC but not of the diffuse type. Stilbenes, hydroxybenzaldehydes, and tyrosols showed inverse associations against both types. Table 2 summarizes the results presented in Figure 2, Figure 3 and Figure 4.

## 4. Discussion

In the current case-control study, our results suggest an inverse association between stilbene and lignan intake and GC risk. We found no overall association between total phenolic acid and other polyphenol classes’ intake and this type of cancer. However, the intake of hydroxybenzaldehydes, hydroxycoumarins, and tyrosols was inversely associated with GC.

By anatomical subsite, no substantial differences were found compared to total GC risk. By histological subtype, the intake of lignans was inversely associated with diffuse GC and that of hydroxybenzaldehydes, tyrosols, and stilbenes with both diffuse and intestinal GC. We found a direct association between hydroxycinnamic acids, phenolic acids, alkylphenols, methoxyphenol, and other polyphenol subclass intake and intestinal GC risk.

There are hardly any epidemiological studies in which the association between these classes of PLPs and GC has been assessed [25,26,27]. Nevertheless, our results are plausible given the results from in vitro and in vivo studies. Different mechanisms by which PLPs could play a role in the prevention or treatment of GC have been described in these studies.

Regarding the stilbene family, both resveratrol and pterostilbene have shown protective effects against GC. Resveratrol has demonstrated the ability to inhibit the growth of human GC cells by induction of apoptosis, increasing the cell load in the G0/G1 phase, and decreasing the proportion of cells in the S and G2/M phases [28]. Resveratrol has also been suggested to show antimicrobial activity through inhibition of the growth of *Helicobacter pylori* by suppressing inducible NO synthase (iNOS), interleukin-8 (IL-8), and Nuclear factor-κappa B (NF-κB) and by activating the factor erythroid 2-related factor 2/Heme oxygenase 1 (Nrf2/HO-1) pathway [29,30]. Pterostilbene has been shown to induce apoptosis in GC cells through activation of the caspase cascade via the mitochondrial pathway, through modification of the cell cycle progress, and through changes in several cycle-regulating proteins [31]. Pterostilbene has higher bioavailability and bioactivity than resveratrol, but its role as a key player in gastric carcinogenesis has been less widely studied [32].

In line with our results, a cohort study showed an inverse association between lignan intake and the risk of gastroesophageal carcinoma [26]. Another case-control study that specifically evaluated GC showed consistent results [25]. This family includes arctigenin, which induces cell cycle arrest and apoptosis [33]. Moreover, schisandrin B can inhibit proliferation and aberrant mitosis by the downregulation of cyclin D1 mRNA expression [34]. Schisantherin A induced cell apoptosis and cell cycle arrest at G2/M phase, inhibited cell migration, induced reactive oxygen species (ROS)dependent Jun N-terminal kinase (JNK) phosphorylation with higher ROS production, and suppressed the expression of Nrf2 in in vitro studies [35].

No previous study has reported an inverse association between hydroxybenzaldehyde intake and GC in particular. However, antimutagenic, anticlastogenic, and anticancer properties have been attributed to vanillin, a type of hydroxybenzaldehyde [36].

Hydroxycoumarins have shown protective effects on gastric cells: esculin conferred significant antioxidant and gastroprotective activity and led to a reduction in gastric injury by inhibition of Nf-κB activation, endogenous prostaglandin and nitric oxide synthesis, and opening of the adenosine triphosphate-sensitive potassium channel (K ATP) [37,38]; esculetin exhibited antiproliferative effects against GC cells through inhibition of the insulin-like growth factor 1 (IGF-1)/ phosphoinositide-3-kinase (PI3K)/ Protein kinase B (Akt ) signaling pathway and induced their apoptosis by a mechanism dependent on caspase activation [39,40,41]; 4-hydroxycoumarin has also shown effects of inhibition of cell proliferation in GC [42].

In the tyrosol family, oleuropein, hydroxytyrosol, and tyrosol have shown antioxidant properties by preventing gastric oxidative damage and improving total antioxidant capacity and cell membrane integrity [43,44]. Furthermore, they have been related to antibacterial actions against *H. pylori* [45]. In this same family, oleuropein-aglycone mono-aldehyde (3,4-DHPEA-EA) and oleuropein-aglycone di-aldehyde (3,4-DHPEA-EDA) have shown antioxidant activity as well induction of apoptosis in tumor cell lines, although this has not been specifically studied in GC [46].

By anatomical location, our results showed no differences with respect to total GC. Cardia GC is more strongly related to obesity, while non-cardia type is more closely related to *H. pylori*; nevertheless, both locations have been associated with *H. pylori* [47,48]. The potential beneficial role of PLPs against this infection have been already discussed.

Regarding the histological subtype, hydroxycinnamic acids, phenolic acids class, alkylphenols, methoxyphenol, and other polyphenol subclasses have shown a direct association with the risk of intestinal GC. This type of GC is often related to environmental factors, diet, and lifestyle, so that our results may partly explain the observed associations between diet and GC [48]. The main source of these PLPs in our study was coffee. It has been found that coffee could potentially increase the risk of GC, although this association has been attributed to the residual confounding effect of tobacco [49,50]. The evidence is still inconsistent.

Several major sources of PLPs have been more studied than the PLPs themselves. stilbenes, hydroxybenzaldehydes, and hydroxybenzoic acids are mainly found in red wine, hydroxycoumarins in beer and wine, and tyrosols and hydroxyphenylacetic acids in olives and olive oil. Nevertheless, the role of wine consumption in gastric carcinogenesis is a controversial topic due to its alcohol content, which is a probable risk factor for GC [51,52]. According to the World Cancer Research Fund [53], the consumption of approximately 30 g or more alcohol per day increases the risk of GC. Despite the fact that excessive alcohol consumption is related to an increased risk of GC, in this study, 59.2% of the controls and 45.6% of the cases showed a daily alcohol intake of 0 and 0–12 g/day. In spite of this, wine has received attention for its hypothesized anticarcinogenic properties. These effects have been attributed to its PLP contents, more concretely to resveratrol, the most widely studied stilbene but also to PLPs from other classes (such as flavonoids or phenolic acids) with which they seem to interact [54,55].

Olive oil has a protective effect against various cancers, including GC, and this property has been attributed to its high PLP content, which supports our results [56,57].

Foods such as olive oil are found within the Mediterranean diet pattern. The Mediterranean diet is characterized by a high intake of vegetables, fruits, legumes, whole-grain cereals, nuts, olive oil; a moderate intake of fish and dairy products; and a low intake of red meat. The properties of this dietary pattern have been attributed—at least partially—to its richness in antioxidants and polyphenols. It has been previously observed that a higher adherence to Mediterranean dietary patterns could reduce the risk of GC [58,59].

We acknowledge that our study may have some limitations. First and given the case-control design of the study, a possible recall bias in the dietary assessment could have led to a misclassification of the exposure. Moreover, self-reported dietary information may have led to some misclassification. However, this misclassification might have been partly reduced because we used a validated FFQ. In addition, the aglycone content in food was estimated without taking into account the losses during cooking, since the retention factors were considered. However, this information is not fully complete for most PLPs, and it may mainly affect to cooked vegetables, which are not large contributors to total phenolics [60]. Furthermore, factors related to climatic stress, geography, and storage conditions can influence the content of polyphenols in food [61,62]. Thus, the heterogeneity in the PLP content of foods grown in different soils, in storage, and in the dietary pattern in each country may explain part of the variability of between the results from studies. [63]. Another important bias related to self-reported dietary intake is the social desirability bias, which can lead to underestimation the alcohol consumption [64]. Additionally, PLPs are extensively metabolized within the human body after consumption, affecting the bioavailability of PLP [65]. In addition, dietary PLPs are consumed simultaneously with other nutrients and compounds. It has been hypothesized that the effects of PLPs may not be explained by a single mechanism of action but rather from many complementary actions of various molecular, biochemical, and physiological pathways and from the additive and synergistic interactions with other phytochemicals [66,67]. Furthermore, our analyses were adjusted for potential confounding variables, but the possible confounding or interactions with other nutrients/compounds cannot be totally ruled out. Finally, it must also be considered that we have assessed classes or subclasses and not individual PLPs. Individual PLPs in the same subclass may have very different bioactivities. Therefore, important associations for individual compounds may have been missed.

On the other hand, our study also shows some strengths. First, we included incident and histologically confirmed cases, and we report our results by histological subtype and anatomical subsite of the tumor. Controls were recruited via random selection from the general population in order to reduce a potential selection bias. Second, our database was built including all the available information about polyphenol contents in Phenol-Explorer, with a mix of extracted data from chromatography, chromatography after hydrolysis data, information from a validated FFQ, and information on a wide range of potential confounders related to GC. This provides a higher reliability from the viewpoint of nutritional epidemiology. Third, PLP intake values were adjusted using the residual method, and the models were adjusted for vegetable and energy intakes, making it sometimes difficult to obtain significant associations between PLP intake and GC. Models were calculated in quartiles and log2 to facilitate the comparison with other studies. Moreover, we have collected a wide array of potential confounders that have been included in the statistical models, which reduces residual confounding and other potential biases. Finally, to our knowledge, this is the first study in which the association between all these families of PLPs and GC has been addressed, including anatomical and histological information. This lack of epidemiologic studies precluded us from comparing our results with others.

## 5. Conclusions

Our results suggest a potential beneficial role of stilbenes, lignans, hydroxybenzaldehydes, hydroxycoumarins, and tyrosols against GC. Our results can only be supported by in vivo and in vitro studies or indirectly by studies based on the PLP food sources. Given the identified gap in epidemiological studies regarding this topic, more prospective research is warranted.

## Figures and Tables

**Table 1:** Characteristics of controls cases of gastric cancer, also by anatomical and histological type.

Variables		Controls	Cases							
			Total		By Anatomical Subtypes			By Histological Subtypes		
		(*n* = 2700)	(*n* = 329)	*p*-Value ^1^	Cardia (*n* = 84)	Non-Cardia (*n* = 238)	*p*-Value ^2^	Intestinal (*n* = 122)	Diffuse (*n* = 75)	*p*-Value ^3^
Age (year) mean (SE)		63.5 (0.2)	65.4 (0.7)	0.001	63.4 (1.3)	66.1 (0.8)	0.036	69.5 (1.0)	61.8 (1.6)	0.000
Sex (men, %)		1522 (56.4)	239 (72.6)	0.000	77 (91.7)	156 (65.6)	0.000	87 (70.7)	45 (60.0)	0.120
Socioeconomic status	High (%)	448 (16.6)	27 (8.2)	0.000	9 (10.7)	18 (7.6)	0.679	8 (6.6)	8 (10.7)	0.372
	Medium (%)	1361 (50.4)	146 (44.4)		38 (45.2)	103 (43.3)		49 (40.2)	34 (45.3)	
	Low (%)	891 (33.0)	156 (47.4)		37 (44.1)	117(49.1)		65 (53.3)	33 (44.0)	
Smoking status (%)	yes	1531 (56.7)	201 (61.1)	0.138	65 (77.4)	130 (54.6)	0.000	60 (49.2)	45 (60.0)	0.229
	no	1169 (43.3)	128 (38.9)		19 (22.6)	108 (45.4)		62 (50.8)	30 (40.0)	
GC family history (%)	yes	170 (6.3)	53 (16.1)	0.000	11 (13.0)	40 (16.8)	0.423	27 (22.1)	13 (17.3)	0.432
	no	2530 (93.7)	276 (83.9)		73 (87.0)	198 (83.2)		95 (77.9)	62 (82.7)	
Physical activity (MET-h/week)	<8	1374 (50.9)	201 (61.1)	0.000	50 (59.5)	147 (61.8)	0.717	66 (54.1)	48 (64.0)	0.187
	≥8	1326 (49.1)	128 (38.9)		34 (40.5)	91 (38.2)		56 (45.9)	27 (36.0)	
Body mass index (kg/m^2^)	≤25	1026 (38.0)	103 (31.3)	0.057	21 (25.0)	80 (33.6)	0.135	41 (33.6)	32 (42.7)	0.415
	>25–30	1129 (41.8)	150 (45.6)		37 (44.0)	110 (46.2)		59 (48.4)	31 (41.3)	
	≥30	545 (20.2)	76 (23.1)		26 (31.0)	48 (20.2)		22 (18.0)	12 (16.0)	
Alcohol consumption (g/day)	0	418 (15.5)	47 (14.3)	0.000	8 (9.5)	39 (16.4)	0.002	22 (18.0)	13 (17.3)	0.592
	<12	1179 (43.7)	103 (31.3)		16 (19.0)	84 (35.3)		40 (32.8)	27 (36.0)	
	12–47	787 (29.1)	101 (30.7)		34 (40.5)	66 (27.7)		30 (24.6)	22 (29.4)	
	>47	316 (11.7)	78 (23.7)		26 (31.0)	49 (20.6)		30 (24.6)	13 (17.3)	
Vegetables total intake (g/d), mean (SE)		191.3 (2.4)	180.8 (7.0)	0.112	184.8 (18.3)	177.9 (7.0)	0.821	189.3 (170.1)	185.6 (13.9)	0.626
Red meat intake (g/d), mean (SE)		64.0 (0.8)	84.4 (2.9)	0.000	97.50 (6.5)	80.10 (3.3)	0.006	84.6 (4.6)	73.1 (5.2)	0.143
Sodium intake (mg/d), mean (SE)		3008.6 (24.0)	3529.3 (86.3)	0.000	3758.6 (200.7)	3443.9 (94.9)	0.175	3403.2 (144.6)	3821.4 (187.7)	0.044
Total phenolic acid intake (mg/d), mean (SE)		166.5 (2.0)	170.7 (5.4)	0.233	191.0 (12.2)	161.1 (5.8)	0.023	178.4 (10.3)	164.5 (10.6)	0.667
Total stilbene intake (mg/d), mean (SE)		1.9 (0.1)	1.67 (0.2)	0.022	2.4 (0.4)	1.4 (0.2)	0.061	1.6 (0.3)	1.3 (0.3)	0.719
Total lignan intake (mg/d), mean (SD)		2.7 (1.7)	2.5 (1.4)	0.085	2.5 (1.3)	2.5 (1.4)	0.712	2.7 (1.5)	2.4 (1.3)	0.260
Total other polyphenol intake (mg/d), mean (SE)		16.4 (0.3)	16.3 (0.9)	0.844	16.1 (1.1)	15.3 (1.3)	0.395	15.3 (1.3)	18.8 (2.6)	0.305

^1^ Differences in categorical and continuous variables between cases and controls using the Pearson chi square test (χ^2^) and ANOVA or Kruskal–Wallis tests, respectively. ^2^ Differences in categorical and continuous variables between cardia and non-cardia groups using the Pearson chi square test (χ^2^) and ANOVA or Kruskal-Wallis tests, respectively. ^3^ Differences in categorical and continuous variables between intestinal and diffuse groups using the Pearson chi square test (χ^2^) and ANOVA or Kruskal-Wallis tests, respectively. MET, metabolic equivalent task.

**Table 2:** Associations of polyphenols (PLPs) with gastric cancer (GC), average PLP consumption (mg/day) and percentage of PLP daily intake in all subjects of the study sorted by the three most consumed foods.

PLP Classes		Total	Anatomical		Histological		PLP Intake mg/day ± SD	Foods with Highest Contribution in All Cases and Controls		
			Cardia	Non-cardia	Intestinal	Diffuse		First (%)	Second (%)	Third (%)
Phenolic acids	Hydroxybenzoic acids	↓	↓	↓	↓	↑	15.73 ± 11.72	Swiss chard (24.4)	Wine (Red) (22.3)	Nuts (14.2)
	Hydroxycinnamic acids	↑	↑	↑	↑ *	↓	150.51 ± 99.41	Coffee (45)	Coffee (decaffeinated) (25)	Apple (5.3)
	Hydroxyphenylacetic acids	↓	↓ *	↓	↓	↑	0.71 ± 1.13	Olives (83.5)	Wine (Red) (11.12)	Beer (Ale/Regular) (3.8)
	Phenolic acids (class)	↑	↑	↑	↑ *	↓				
Stilbenes	Stilbenes	↓ *	↓	↓ *	↓	↓	1.86 ± 3.06	Wine (Red) (92.2)	Wine (Rosé) (3.8)	Grape (2)
Lignans	Lignans	↓ *	↓ *	↓ *	↓	↓ *	2.71 ± 1.70	*Brassica oleracea* (22.4)	Green bean (17.4)	Orange tangerine (11.9)
Other polyphenols	Alkylmethoxyphenols	↑	↑	↑	↑	↑	0.74 ± 0.92	Coffee (70.4)	Coffee (decaffeinated (22.3)	Beer (Ale/Regular) (7.3)
	Alkylphenols	↑	↓	↑	↑ *	↓	0.09 ± 0.09	Coffee (98)	Beer (1.8)	Cocoa powder (0.2)
	Hydroxybenzaldehydes	↓ *	↓ *	↓ *	↓ *	↓ *	0.38 ± 0.63	Wine (Red) (92.7)	Wine (Rosé) (2.8)	Beer (Ale/Regular) (18)
	Hydroxycoumarins	↓ *	↓ *	↓ *	↓	↓	0.07 ± 0.14	Beer (Ale/Regular) (55.2)	Wine (Rosé) (44)	Sherry (0.7)
	Methoxyphenol	↑	↑	↑	↑ *	↓	0.10 ± 0.13	Coffee (100)		
	Tyrosols	↓ *	↓ *	↓ *	↓ *	↓	14.06 ± 16.18	Olives (58.2)	Olive oil (26.4)	Wine (Red) (12.8)
	Other polyphenols (subclass) ^a^	↑ *	↑	↑ *	↑*	↓	0.89 ± 0.90	Coffee (36.9)	Orange juice (23.2)	Other juice (13.7)
	Other polyphenols (class)	↑	↑	↑	↑*	↓				

Other polyphenols (subclass) ^a^ This subclass contains the intake of arbutin, catechol, coumestrol, phenol, phlorin, and pyrogallol. ↑ increased risk; ↓ decreased risk; * statistically significant.

## Supplementary Materials

The following are available online at https://www.mdpi.com/2072-6643/12/11/3281/s1, Figure S1: Quartiles distribution, OR (95% CIs), and Log2 (95% CIs) of all gastric cancer cases according to subclass of polyphenol intakes in the MCC-Spain study by anatomical site and histological type.
