# Supplementary material for: Association between Polyphenol Intake and Gastric Cancer Risk by Anatomic and Histologic Subtypes: MCC-Spain
Source: Nutrients. 2020 Oct 26;12(11):3281. doi: 10.3390/nu12113281 (PMC7692577; doi:10.3390/nu12113281)
Supplement: Supplementary file 1 [file nutrients-12-03281-s001.pdf]

Table S1. Quartiles distribution, OR (95% CIs) and Log2 (95% CIs) of all gastric cancer cases according to subclass of polyphenols intakes in the MCC-Spain study by anatomical site and histological type.

Polyphenol subclasses		Quartil Intake (mg/d)		*Total Cases*			*Cardias*			*No Cardias*			*Intestinal*			*Diffuse*		
				Cases	*OR CI (95%)*	*Continuous (log2)*	*Cases*	*OR CI (95%)*	*Continuous (log2)*	*Cases*	*OR CI (95%)*	*Continuous (log2)*	*Cases*	*OR CI (95%)*	*Continuous (log2)*	*Cases*	*OR CI (95%)*	*Continuous (log2)*
**Phenolic acids**	**Hydroxybenzoic acids**	Q1	<8.1	97	1		24	1		72	1		38	1		21	1	
		Q2	8.1-12.3	97	1.26 (0.91-1.75)		21	1.29 (0.68-2.47)		74	1.26 (0.87-1.82)		32	1.06 (0.62-1.82		21	1.50 (0.78-2.88)	
		Q3	12.3-17.19	75	1.05 (0.74-1.51)	0.90 (0.80-1.00)	21	1.32 (0.67-2.58)	0.98 (0.80-1.20)	51	0.97 (0.64-1.47)	0.88 (0.77-0.99)	34	1.30 (0.75-2.25)	0.88 (0.73-1.06)	16	1.26 (0.62-2.57)	1.10 (0.87-1.40)
		Q4	>17.19	60	0.69 (0.47-1.02)		18	0.75 (0.37-1.53)		41	0.71 (0.45-1.10)		19	0.62 (0.33-1.16)		17	1.16 (0.56-2.38)	
		p-tend		0.061			0.487			0.095			0.309			0.764		
	**Hydroxycinnamic acids**	Q1	<87.68	77	1		15	1		61	1		29	1		19	1	
		Q2	87.68-130.43	92	1.26 (0.89-1.78)		24	1.66 (0.83-3.32)		67	1.18 (0.80-1.73)		25	1.04 (0.58-1.89)		25	1.35 (0.73-2.54)	
		Q3	130.43-188.98	73	1.10 (0.76-1.58)	1.04 (0.92-1.19)	21	1.62 (0.80-3.32)	1.21 (0.94-1.57)	51	0.97 (0.64-1.47)	0.97 (0.85-1.13)	31	1.44 (0.82-2.53)	1.30 (1.04-1.64)	15	0.93 (0.46-1.89)	0.83 (0.67-1.03)
		Q4	>188.98	87	1.40 (0.98-2.00)		24	1.82 (0.90-3.69)		59	1.23 (0.82-1.85)		38	2.21 (1.25-3.90)		16	0.87 (0.43-1.79)	
		p-tend		0.133			0.138			0.512			0.003			0.502		
	**Hydroxyphenylacetic acids**	Q1	<0.122	113	1		36	1		74	1		44	1		23	1	
		Q2	0.12-0.32	71	0.81 (0.57-1.15)		13	0.49 (0.24-0.98)		56	0.95 (0.64-1.41)		25	0.67 (0.38-1.17)		16	1.01 (0.51-2.03)	
		Q3	0.32-0.66	70	0.85 (0.59-1.21)	0.94 (0.87-1.02)	19	0.74 (0.39-1.39)	0.85 (0.73-0.98)	51	0.93 (0.61-1.40)	0.96 (0.89-1.06)	27	0.91 (0.51-1.60)	0.91 (0.81-1.03)	14	0.91 (0.44-1.89)	0.96 (0.83-1.10)
		Q4	>0.66	75	0.78 (0.55-1.10)		16	0.50 (0.26-0.96)		57	0.80 (0.61-1.33)		27	0.76 (0.44-1.31)		22	1.10 (0.59-2.09)	
		p-tend		0.197			0.073			0.603			0.491			0.821		
	**Phenolic acids (class)**	Q1	<99.72	82	1		17	1		64	1		33	1		20	1	
		Q2	99.72-145.69	85	1.06 (0.75-1.49)		22	1.33 (0.67-2.63)		62	1.01 (0.69-1.48)		22	0.78 (0.43-1.42)		23	1.18 (0.63-2.21)	
		Q3	145.69-204.59	78	1.11 (0.77-1.58)	1.01 (0.88-1.17)	21	1.40 (0.70-2.80)	1.19 (0.90-1.57)	56	1.04 (0.69-1.55)	0.94 (0.80-1.11)	30	1.26 (0.72-2.19)	1.30 (1.02-1.67)	17	1.07 (0.54-2.11)	0.79 (0.62-1.01)
		Q4	>204.59	84	1.26 (0.88-1.80)		24	1.56 (0.78-3.12)		56	1.12 (0.75-1.69)		38	1.99 (1.14-3.45)		15	0.80 (0.39-1.65)	
		p-tend		0.202			0.219			0.580			0.007			0.545		
**Stilbenes**	**Stilbenes**	Q1	<0.1	124	1		31	1		92	1		46	1		30	1	
		Q2	0.1-0.27	82	1.03 (0.73-1.46)		21	1.16 (0.62-2.19)		59	0.90 (0.63-1.39)		34	1.03 (0.60-1.77)		17	1.03 (0.53-2.01)	
		Q3	0.27-0.67	69	0.82 (0.57-1.16)	0.88 (0.83-0.94)	15	0.70 (0.35-1.38)	0.86 (0.78-0.96)	52	0.81 (0.54-1.21)	0.89 (0.83-0.96)	20	0.61 (0.34-1.13)	0.88 (0.80-0.96)	17	1.06 (0.54-2.10)	0.95 (0.83-1.08)
		Q4	>0.67	54	0.47 (0.32-0.69)		17	0.54 (0.28-1.06)		35	0.44 (0.28-0.70)		23	0.59 (0.33-1.07)		11	0.50 (0.23-1.10)	
		p-tend		0.000			0.040			0.001			0.034			0.146		
**Lignans**	**Lignans**	Q1	<1.90	112	1		34	1		77	1		34	1		32	1	
		Q2	1.90-2.69	75	0.78 (0.55-1.10)		16	0.62 (0.32-1.19)		57	0.82 (0.55-1.21)		24	0.64 (0.36-1.16)		19	0.76 (0.41-1.41)	
		Q3	2.69-3.68	85	0.86 (0.61-1.21)	0.79 (0.69-0.91)	21	0.83 (0.44-1.54)	0.84 (0.67-1.07)	62	0.88 (0.59-1.30)	0.77 (0.66-0.90)	37	0.97 (0.56-1.68)	0.87 (0.70-1.08)	14	0.56 (0.28-1.12)	0.72 (0.57-0.90)
		Q4	>3.68	57	0.53 (0.36-0.77)		13	0.45 (0.22-0.93)		42	0.55 (0.35-0.84)		28	0.69 (0.39-1.24)		10	0.34 (0.16-0.74)	
		p-tend		0.003			0.065			0.015			0.470			0.004		
**Other polyphenols**	**Alkylmethoxyphenols**	Q1	<0.14	91	1		24	1		66	1		38	1		18	1	
		Q2	0.14-0.34	62	0.78 (0.54-1.12)		11	0.50 (0.24-1.07)		50	0.87 (0.58-1.31)		16	0.54 (0.29-1.02)		19	1.20 (0.61-2.39)	
		Q3	0.34-1.06	91	1.04 (0.74-1.45)	1.01 (0.95-1.08)	23	0.92 (0.49-1.72)	1.02 (0.90-1.17)	66	1.07 (0.73-1.58)	0.99 (0.93-1.08)	33	1.11 (0.65-1.88)	1.05 (0.94-1.16)	22	1.12 (0.57-2.19)	0.99 (0.87-1.13)
		Q4	>1.06	85	1.12 (0.79-1.59)		26	1.26 (0.68-2.35)		56	1.01 (0.68-1.51)		36	1.47 (0.86-2.50)		16	1.03 (0.50-2.11)	
		p-tend		0.284			0.243			0.715			0.061			0.983		
	**Alkylphenols**	Q1	<0.01	84	1		19	1		64	1		28	1		23	1	
		Q2	0.01-0.06	90	1.06 (0.75-1.48)	1.00 (0.93-1.07)	22	1.00 (0.52-1.93)	0.98 (0.85-1.13)	67	1.06 (0.73-1.55)	1.00 (0.92-1.08)	38	1.55 (0.90-2.66)	1.06 (0.95-1.18)	15	0.59 (0.30-1.19)	0.95 (0.83-1.09)
		Q3	0.06-0.13	73	0.93 (0.64-1.32)		21	0.92 (0.47-1.81)		50	0.87 (0.57-1.31)		22	1.13 (0.61-2.10)		20	0.84 (0.44-1.61)	

		Q4	>0.13	82	1.06 (0.74-1.52)		22	0.95 (0.48-1.87)		57	1.06 (0.70-1.59)		35	2.09 (1.17-3.73)		17	0.68 (0.34-1.36)	
		p-tend		0.914			0.831			0.963			0.040			0.445		
	**Hydroxybenzaldehydes**	Q1	<0.01	129	1		33	1		95	1		49	1		23	1	
		Q2	0.01-0.05	81	0.94 (0.67-1.32)		19	0.89 (0.47-1.70)		60	0.89 (0.60-1.31)		34	1.00 (0.59-1.72)		15	0.77 (0.38-1.52)	
		Q3	0.05-0.13	68	0.73 (0.51-1.04)	0.88 (0.83-0.94)	17	0.70 (0.36-1.34)	0.86 (0.76-0.96)	48	0.69 (0.46-1.03)	0.89 (0.83-0.96)	20	0.61 (0.33-1.11)	0.88 (0.80-0.97)	20	0.92 (0.47-1.79)	0.89 (0.78-1.00)
		Q4	>0.13	51	0.41 (0.28-0.61)		15	0.41 (0.21-0.82)		35	0.41 (0.26-0.64)		20	0.50 (0.27-0.91)		17	0.39 (0.17-0.87)	
		p-tend		0.000			0.010			0.000			0.009			0.047		
	**Hydroxycoumarins**	Q1	<0.001	130	1		35	1		92	1		49	1		31	1	
		Q2	0.001-0.012	83	0.99 (0.70-1.39)		20	1.02 (0.54-1.92)		61	0.93 (0.63-1.37)		33	1.02 (0.59-1.75)		15	0.80 (0.40-1.59)	
		Q3	0.012-0.034	54	0.67 (0.46-0.98)	0.84 (0.79-0.90)	13	0.57 (0.29-1.16)	0.81 (0.72-0.91)	39	0.66 (0.43-1.03)	0.86 (0.80-0.93)	18	0.72 (0.38-1.36)	0.85 (0.77-0.94)	13	0.68 (0.33-1.40)	0.83 (0.73-0.93)
		Q4	>0.034	62	0.49 (0.34-0.71)		16	0.39 (0.20-0.77)		46	0.55 (0.36-0.84)		23	0.60 (0.33-1.08)		16	0.59 (0.29-1.18)	
		p-tend		0.000			0.003			0.002			0.058			0.119		
	**Methoxyphenols**	Q1	<0.02	78	1		18	1		59	1		33	1		18	1	
		Q2	0.02-0.04	75	1.07 (0.75-1.54)		18	1.06 (0.53-2.15)		56	1.06 (0.71-1.59)		19	0.73 (0.40-1.37)		21	1.31 (0.68-2.55)	
		Q3	0.04-0.15	92	1.29 (0.92-1.82)	1.03 (0.97-1.09)	24	1.51 (0.79-2.92)	1.07 (0.95-1.21)	66	1.25 (0.85-1.84)	1.01 (0.94-1.09)	32	1.26 (0.73-2.17)	1.06 (0.97-1.17)	22	1.20 (0.62-2.32)	0.97 (0.86-1.10)
		Q4	>0.15	84	1.35 (0.95-1.92)		24	1.73 (0.89-3.39)		57	1.17 (0.77-1.76)		39	1.78 (1.04-3.03)		14	0.93 (0.44-1.93)	
		p-tend		0.057			0.062			0.328			0.013			0.834		
	**Tyrosols**	Q1	<5.61	121	1		36	1		81	1		46	1		25	1	
		Q2	5.61-9.03	83	0.85 (0.61-1.18)		19	0.76 (0.41-1.40)		63	0.91 (0.63-1.33)		28	0.68 (0.40-1.17)		20	1 (0.55-1.97)	
		Q3	9.03-13.82	60	0.63 (0.44-0.90)	0.86 (0.78-0.95)	14	0.56 (0.28-1.10)	0.83 (0.70-0.98)	45	0.66 (0.44-1.00)	0.87 (0.78-0.97)	27	0.72 (0.42-1.24)	0.85 (0.73-0.98)	13	0.66 (0.32-1.36)	0.91 (0.76-1.09)
		Q4	>13.82	65	0.56 (0.39-0.80)		15	0.45 (0.23-0.88)		49	0.63 (0.42-0.94)		22	0.50 (0.29-0.90)		17	0.72 (0.37-1.40)	
		p-tend		0.000			0.011			0.009			0.027			0.205		
	**Other polyphenols (subclass)**	Q1	<0.5	87	1		23	1		61	1		31	1		22	1	
		Q2	0.5-0.78	68	1.02 (0.72-1.48)		17	1.08 (0.55-2.13)		51	1.06 (0.71-1.61)		24	1.01 (0.56-1.83)		17	1.05 (0.54-2.06)	
		Q3	0.78-1.2	85	1.31 (0.93-1.86)	1.14 (1.03-1.27)	21	1.33 (0.69-2.58)	1.13 (0.94-1.38)	63	1.37 (0.92-2.03)	1.14 (1.00-1.29)	33	1.58 (0.91-2.75)	1.25 (1.05-1.50)	21	1.37 (0.72-2.61)	1.01 (0.85-1.21)
		Q4	>1.2	89	1.49 (1.06-2.10)		23	1.50 (0.80-2.84)		63	1.50 (1.01-2.23)		35	1.98 (1.14-3.45)		15	0.87 (0.44-1.74)	
		p-tend		0.010			0.172			0.023			0.006			0.959		
	**Other polyphenols (class)**	Q1	<6.73	117	1		33	1		80	1		44	1		27	1	
		Q2	6.73-10.27	81	0.86 (0.62-1.19)		18	0.71 (0.39-1.32)		62	0.93 (0.64-1.35)		30	0.77 (0.46-1.30)		15	0.93 (0.49-1.76)	
		Q3	10.27-15.04	63	0.64 (0.45-0.92)	0.87 (0.78-0.97)	17	0.58 (0.30-1.11)	0.83 (0.69-1.01)	46	0.68 (0.45-1.02)	0.88 (0.78-0.99)	24	0.72 (0.41-1.25)	0.89 (0.75-1.06)	16	0.68 (0.33-1.36)	0.89 (0.73-1.08)
		Q4	>15.04	68	0.56 (0.39-0.80)		16	0.44 (0.23-0.86)		50	0.62 (0.41-0.92)		25	0.56 (0.32-0.99)		17	0.64 (0.32-1.25)	
		p-tend		0.000			0.012			0.008			0.046			0.133		

Adjusted multivariate log2 and 95% confidence intervals (CI) for age. socioeconomic status. alcohol consumption. smoking status. salt intake. body mass index. physical activity. first-degree family history. red meat intake. vegetables intake. and total energy intake including the study area as a random effect term. OR, odd ratio; CI, confidence intervals; MCC-Spain, multi-case-control study Spain.
